# Enhancing Median Nerve Regeneration: A Literature Review of Electrical Stimulation and Adjunctive Strategies

**DOI:** 10.7759/cureus.117854

**Published:** 2026-10-13

**Authors:** Sophia Kermet, Luke Hudson, Brenda Hranec, Marley Homandberg, James Kotick

**Affiliations:** 1 College of Medicine, Florida State University, Tallahassee, USA; 2 Plastic Surgery, Envision Plastic and Reconstructive Surgery, Sarasota, USA

**Keywords:** axonal regeneration, electrical stimulation, median nerve injury, nerve repair, nerve repair techniques, peripheral nerve regeneration

## Abstract

Peripheral nerve injuries continue to be a cause of long-term disability despite advances in surgical repair techniques. Complete recovery of nerve function is rarely seen, especially in mixed motor and sensory innervation. Injuries involving the median nerve are particularly debilitating due to their essential role in hand sensation, grip strength, and fine motor control. This review aims to examine the current standard of care for median nerve repair and examine the emerging role of electrical stimulation (ES) as an adjunctive therapy within a multimodal reconstructive framework. Current surgical management includes tension-free neurorrhaphy for amenable nerve gaps, interpositional nerve grafting for larger defects, and nerve conduits in selected short-gap injuries. The exact approach for each case is often dictated by the distance between damaged nerve endings. While current standards reliably restore anatomic continuity, they are limited by slow axonal regeneration, prolonged denervation, and Schwann cell senescence. Flaps can improve vascularized soft tissue coverage in the damaged area. Perfusion to the regenerating nerve increases axonal spread. ES in conjunction with standard treatment has been shown to enhance axonal outgrowth, accelerate target reinnervation, and promote pro-regenerative signaling in neurons and Schwann cells, particularly when applied early after repair and combined with structural reconstruction.

## Introduction and background

Peripheral nerve injuries remain a significant source of long-term morbidity despite substantial advances in microsurgical repair. Although modern techniques reliably restore anatomic continuity, functional recovery is often incomplete. In particular, mixed motor and sensory nerves of the upper extremity often result in substantial functional deficits after recovery [1]. Among these, median nerve injuries are especially debilitating [2]. Loss of median nerve function results in deficits apparent in precision grip, sensation to the first three digits, and wrist flexion. Even after the median nerve is surgically repaired, patients frequently experience persistent deficits. Functional limitations seen post-repair carry substantial social and economic consequences, particularly for working-age individuals whose livelihoods are dependent on manual dexterity.

Tension-free end-to-end neurorrhaphy and interpositional nerve grafting remain the standard approaches to peripheral nerve repair [3], yet by addressing the structural component of the injury primarily, they do not fully recreate the complex biological environment required for successful regeneration [4]. Traumatic nerve injuries are frequently accompanied by localized ischemia, inflammation, fibrosis, and disruption of the native extracellular matrix (ECM). These additional factors often hinder axonal regeneration through mechanisms such as impaired Schwann cell function, decreased growth factor availability, and fibrosis [5]. As regeneration proceeds slowly, typically at a rate of 1-3 mm per day, prolonged denervation further exacerbates muscle atrophy and Schwann cell senescence, progressively narrowing the therapeutic window for meaningful recovery [6].

In this review, we examine the biological rationale and clinical evidence supporting the use of electrical stimulation (ES) and complementary adjunctive strategies in median nerve repair. We draw on principles of peripheral nerve anatomy, surgical reconstruction, vascularized soft tissue coverage, and bioelectric modulation. This review is necessary to broadly establish the role of ES in peripheral nerve injury, reconstruction, and regeneration, with a focus on clinical context, including high-voltage electrical burns. The synthesis may guide future clinical uses and research.

## Review

Methods

A structured search was conducted on PubMed, Google Scholar, and the Excerpta Medica database (Embase) on May 24, 2026, with no restriction on publication year. Search terms included “median nerve,” “peripheral nerve repair,” “electrical stimulation,” “peripheral nerve regeneration,” “axon regeneration,” “neurorrhaphy,” and “Functional recovery.” Studies were included if they focused on median nerve injury, repair, or regeneration or evaluated ES for nerve regeneration in clinical, animal, and in vitro studies. Additionally, English full texts were required for inclusion in this review. Studies were excluded if they were unrelated to peripheral nerve regeneration or electrical stimulation, focused on central nervous system injury only, or non-peer-reviewed publications. Relevant information was extracted, such as study design, nerve injury model, surgical or adjunctive intervention, and measured outcomes.

Principles of peripheral nerve repair and adjunctive therapies 

Surgical repair of peripheral nerve injuries presents a host of diverse clinical challenges, from extensive tissue loss as a result of trauma to clean transections. The type, location, severity of the injury, and the gap between nerve endings all dictate the surgical technique chosen for the repair. The salient goal of nerve repair is to preserve nerve function while minimizing tension, scarring, and neuroma formation [7].

In cases of complete transections with a minimal gap between free nerve endings, tension-free end-to-end neurorrhaphy is the gold standard in nerve repair. When there is a gap between nerve stumps too large to approximate without tension, interpositional nerve reconstruction is the standard method of repair [3]. This technique relies on a graft to bridge the gap between the two nerve endings. The most common approach is autografting, where a section of the patient’s own sensory nerves is harvested to bridge the endings [3]. Autografting provides an ideal scaffold for axonal regeneration, but the sensory deficit at the donor site is important to consider. Allografting circumvents this drawback by utilizing cadaver nerves as the donor material. Because the foreign nerve source is immunogenic, temporary immunosuppression is often employed to prevent rejection of the graft. Unlike solid-organ transplantation, however, nerve allografting uses the graft only as a temporary guide that host tissue is built upon. As such, immunosuppressive therapy is only required until native axons grow past the foreign tissue [8].

Alternative methods to immunosuppression come in the form of pre-grafting treatment of the donor material. Freeze-thaw processing, where allografts are subjected to repeated freezing and thawing cycles that lyse antigenic cells while preserving the ECM, is one such technique that has been shown in animal models to eliminate the need for immunosuppression while preserving graft function [9]. Chemical detergent decellularization strips donor nerve allografts of their cellular components, making the graft immunologically inert. Left behind is a protein network through which the host axon can grow [9]. The resulting decellularized human nerve allografts are US FDA-approved for nerve gaps up to 70 mm, making them a practical option for longer defects such as those of the median nerve [10]. Both of these pre-treatment methods allow for transplant without systemic immunosuppression and serve as promising options when autografts are not available.

Tubularized nerve reconstruction is the placement of a conduit to shelter the gap between transected nerve endings. The conduit is usually either a bioabsorbable material, such as polyglycolic acid or collagen, or a biological conduit such as an autologous vein [11]. By creating a protective environment, the conduit facilitates axonal growth directed towards the opposite free end. This technique demonstrates the greatest efficacy in injuries with gaps less than 3 cm with clean, well-aligned, and adequately vascularized nerve stumps [11].

The success of peripheral nerve regeneration, particularly for large-gap injury, is dictated by the degree to which the surrounding tissue is vascularized. Intrinsic vessels within the nerve endoneurium are highly susceptible to ischemia during hypoperfusion [5]. This places a particularly high importance on vascularization to ensure continual blood flow to the growing nerve. The hierarchical organization of the median nerve and the distribution of its epineurial, perineurial, and endoneurial vasculature are shown in Figure 1.

**Figure 1:**
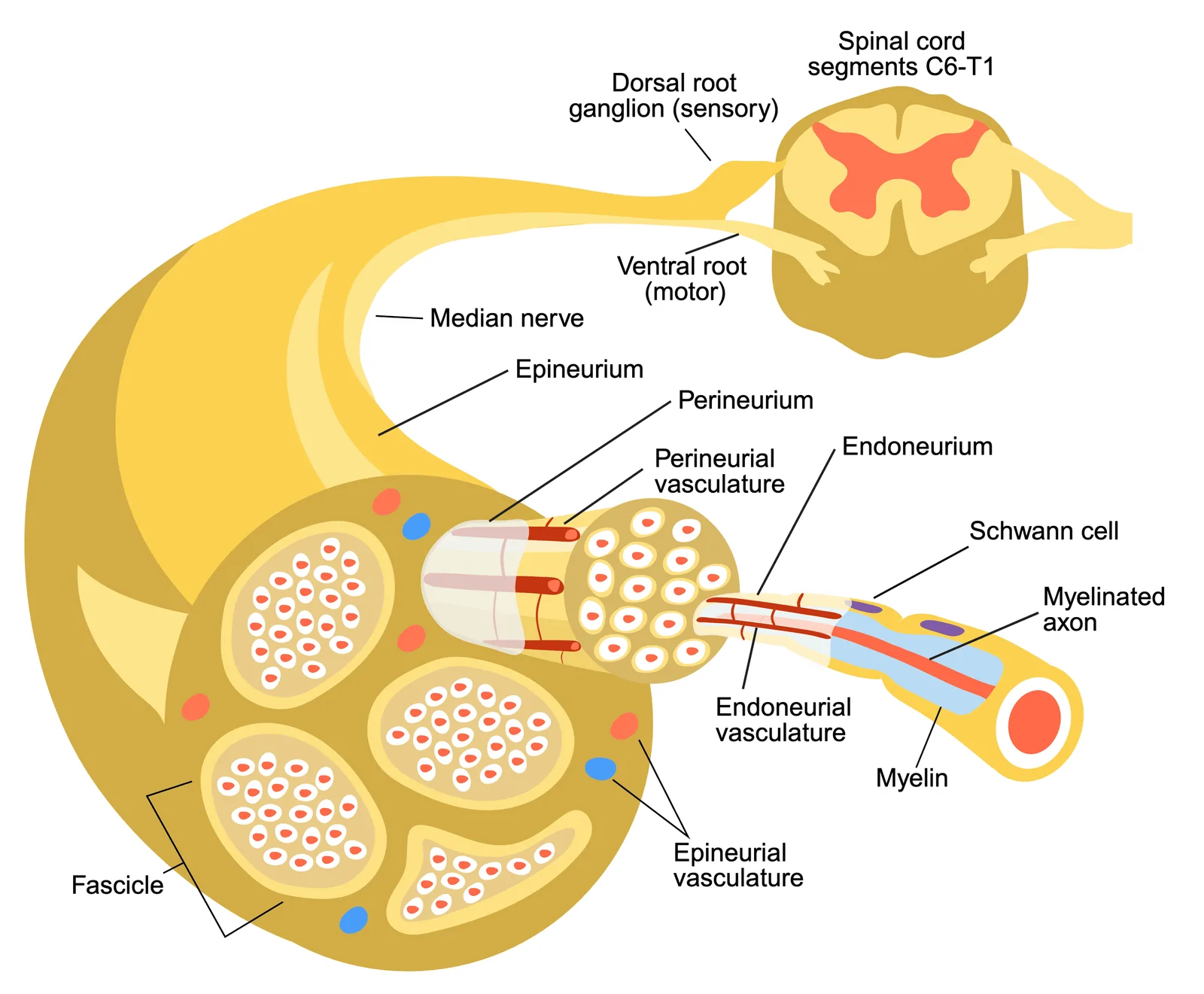
Architecture and intraneural vasculature of the median nerve Myelinated nerve fibers are surrounded by endoneurium and grouped into fascicles bounded by perineurium. Fascicles and their associated vessels are enclosed by the epineurium. Epineurial, perineurial, and endoneurial vasculature are labeled separately to illustrate the intraneural blood supply vulnerable to disruption after injury. The nerve is shown arising from spinal cord segments C6-T1; brachial plexus contributions are not depicted. Labels: median nerve, dorsal root ganglion (sensory), ventral root (motor), spinal cord segments C6-T1, epineurium, perineurium, endoneurium, fascicle, myelinated axon, myelin, Schwann cell, epineurial vasculature, perineurial vasculature, endoneurial vasculature. Created in BioRender. Hudson, L. (2026); https://BioRender.com/457z20q.

During regeneration, angiogenesis often precedes Schwann cell migration and axon growth, highlighting the interdependence of vascular and neural regeneration [4]. Blood vessels also act as a shuttle for Schwann cells to traverse until they reach the site of repair [4].

Surgical intervention can be used to increase the vascularized soft tissue surrounding the damaged nerve. Vascularized tissue transfer using either pedicled or free flaps relocates healthy, perfused tissue into the injured area. This process is particularly useful in extensive wounds, where local tissue is insufficient or significantly damaged [12]. Beyond restoring vascularized soft-tissue coverage, flaps have a secondary purpose of providing mechanical protection to the regenerating nerve. 

Strategies for nerve regeneration 

Advances in microsurgical technique for peripheral nerves have progressed over recent years in an attempt to combat the complexity of mixed-innervation nerve damage, such as the median nerve. However, functional recovery outcomes remain inconsistent. The demand for more reliable and consistent outcomes has driven the development of adjunctive strategies that act on the biology of regeneration rather than on the choice of surgical reconstruction [13]. Whereas the preceding section discussed how gap length and tension dictate the selection of neurorrhaphy, autograft, processed allograft, or conduit, the strategies discussed here address a distinct problem: how to improve directional specificity, preserve the microenvironment during regeneration, and augment axonal regeneration. These aims have motivated the development of multiple distinct modalities. 

The reconstructive constructs introduced above also serve as platforms for biological augmentation. Rather than functioning solely as structural support, both nerve conduits and decellularized allografts can be enriched or combined with adjunctive interventions to enhance the microenvironment they provide (as described above, conduits are indicated for short, well-vascularized gaps and decellularized allografts for longer nerve defects). The focus of current investigation is therefore shifting from what these constructs are to how their regenerative capacity can be actively enhanced. 

Shortcomings in autografts have continued the search for biological supplementation adjacent to surgery. Recent evidence points to the benefit of combining isolated interventions with ES to elicit a synergistic effect. ES works by promoting pro-growth pathways such as mitogen-activated protein kinase/extracellular signal-regulated kinase (MAPK/ERK) and phosphoinositide 3-kinase/protein kinase B (PI3K/Akt) in neuronal and non-neuronal supportive cells such as Schwann cells [14].

Electrical stimulation 

ES has emerged as a promising adjunctive strategy in peripheral nerve repair when integrated with established surgical and reconstructive principles rather than applied in isolation. Rather than replacing foundational microsurgical techniques such as tension-free coaptation, gap bridging, and adequate soft-tissue coverage, ES functions as a biological amplifier that enhances the intrinsic regenerative capacity of injured nerves. A growing body of experimental evidence demonstrates that ES accelerates axonal outgrowth, improves directional specificity, and shortens the time to functional reinnervation, effects that are particularly critical in long mixed nerves such as the median nerve.

At the cellular level, ES promotes a pro-regenerative phenotype in both neurons and Schwann cells through activity-dependent signaling pathways. Brief, low-frequency ES applied following nerve injury increases intracellular calcium influx, activating downstream signaling cascades including MAPK/ERK and PI3K/Akt, which are essential for neuronal survival and axonal elongation. Activation of these pathways induces a transcriptional shift toward a growth state, characterized by the upregulation of regeneration-associated genes [14].

One of the central mechanisms by which ES enhances nerve regeneration is through the upregulation of neurotrophic factors, particularly brain-derived neurotrophic factor (BDNF), which plays a critical role in axonal elongation and repair [14]. Although nerve growth factor (NGF) and glial cell line-derived neurotrophic factor (GDNF) are well-established contributors to peripheral nerve regeneration, direct evidence linking ES to their upregulation is more limited in the current literature. In contrast, multiple experimental studies demonstrate a robust and reproducible association between ES and increased BDNF expression. Al-Majed et al. [15] showed that one hour of ES resulted in rapid upregulation of BDNF and its receptor tropomyosin receptor kinase B, with mRNA levels increasing threefold and twofold, respectively, within eight hours and peaking at two days with sixfold and fourfold increases. This molecular response correlated with accelerated axonal regeneration across the repair site and improved target reinnervation [15]. These effects are time-dependent, with the greatest benefit observed when ES is applied acutely following nerve repair, resulting in faster axonal crossing of the injury site and earlier target reinnervation [16, 17].

Schwann cells, which are indispensable for peripheral nerve regeneration, also respond robustly to ES. Electrical cues promote Schwann cell proliferation, alignment, and preservation of the bands of Büngner, thereby enhancing axonal guidance across the injury zone [18, 19]. ES further accelerates Wallerian degeneration, facilitating Schwann cell dedifferentiation and proliferation. Koppes et al. [18] demonstrated that electrically prestimulated Schwann cells produced sustained increases in neurite outgrowth, accompanied by an 11-fold increase in NGF secretion that persisted for up to 72 hours following stimulation.

In addition to enhancing acute regeneration, ES has been associated with delayed Schwann cell senescence, counteracting the progressive decline in regenerative capacity observed with prolonged denervation [20, 21]. Elzinga et al. [21] showed that brief ES significantly improved nerve regeneration even after three months of chronic denervation, a stage at which Schwann cells typically undergo atrophy and lose their ability to support axonal regrowth. The underlying mechanism appears to involve sustained expression of the neurotrophic and growth-associated gene programs described above, which otherwise diminish over time in chronically denervated Schwann cells [20].

The regenerative effects of ES are further enhanced when combined with nerve guidance conduits. While conduits provide structural continuity and protect the intraneural microenvironment, ES supplies a bioelectric signal that promotes axonal growth and directional guidance [22]. Preclinical studies utilizing electrically conductive or electrode-integrated conduits demonstrate increased axonal density, accelerated reinnervation, and improved functional recovery compared with conduits alone [22, 23]. Notably, these combined strategies have been shown to restore nerve conduction velocity to approximately 74% of normal values by three months post-repair in rodent models [24], with concurrent increases in myelin thickness and upregulation of neurotrophic factors and neural markers [23]. Mechanistically, this synergy is mediated through activation of pro-regenerative signaling pathways, including MAPK/ERK signaling, which are critical for successful nerve regeneration [23].

This combined approach shows particular promise for peripheral nerve injuries requiring gap reconstruction. Long regeneration distances impose substantial biological challenges, including prolonged axotomy, chronic denervation, deterioration of the distal nerve stump, and target organ atrophy [22, 25]. Although the majority of preclinical evidence has been derived from sciatic nerve models, the underlying principles are applicable to complex mixed nerves such as the median nerve, where fascicular complexity and long regeneration distances increase the risk of axonal misdirection [23]. Studies demonstrating improved directional cell migration and axonal alignment with electrically conductive conduits support the translational relevance of this strategy [23].

In complex injuries, particularly high-voltage electrical burns, restoration of the regenerative environment requires additional consideration of soft-tissue reconstruction. Vascularized flap coverage plays a critical role in mitigating ischemia, inflammation, and fibrosis, while improving perfusion and supporting the metabolic demands of regenerating nerves [26]. Early microsurgical flap coverage, typically within 21 to 27 days post-injury, has been associated with successful limb salvage and functional preservation in electrical burn patients, reducing extremity loss rates from 41.5% to 7% when combined with early debridement, preservation of vital structures, and comprehensive reconstruction [27]. When integrated with ES, flap reconstruction may further enhance outcomes by stabilizing implanted stimulation interfaces and improving tissue conductivity, thereby supporting sustained delivery of electrical signals. ES, in turn, accelerates axonal outgrowth, remyelination, and end-organ reinnervation through elevation of neuronal cyclic adenosine monophosphate (cAMP) and upregulation of neurotrophic factors and growth-associated genes [17]. Although direct clinical data examining combined ES and flap reconstruction remain limited, the biological rationale is compelling, with principles from reconstructive and neuroprosthetic literature supporting their complementary roles in severe nerve injuries requiring both structural support and optimization of the regenerative microenvironment [17].

Collectively, these findings support a multimodal paradigm for peripheral nerve repair in which ES functions as one component of an integrated strategy alongside structural repair and optimized soft-tissue management. This approach is particularly relevant for extensive median nerve injuries, where prolonged denervation and long regeneration distances progressively narrow the therapeutic window for functional recovery. Chronically axotomized neurons lose regenerative capacity, denervated Schwann cells undergo atrophy, and target end organs become irreversibly damaged while axonal regeneration proceeds at only 1-3 mm per day. ES addresses declining neuronal regenerative capacity by elevating cAMP and sustaining growth-associated gene expression, while structural repair provides physical guidance and vascularized soft-tissue coverage optimizes perfusion and reduces inflammation. Together, this integrated strategy maximizes the likelihood of timely and meaningful functional reinnervation in complex injuries requiring gap reconstruction, where biological and anatomical challenges otherwise predispose to poor outcomes [14, 23, 25]. Figure 2 illustrates the repaired nerve during regeneration and the sites at which ES, structural bridging, and preservation of the distal Schwann cell environment each act.

**Figure 2:**
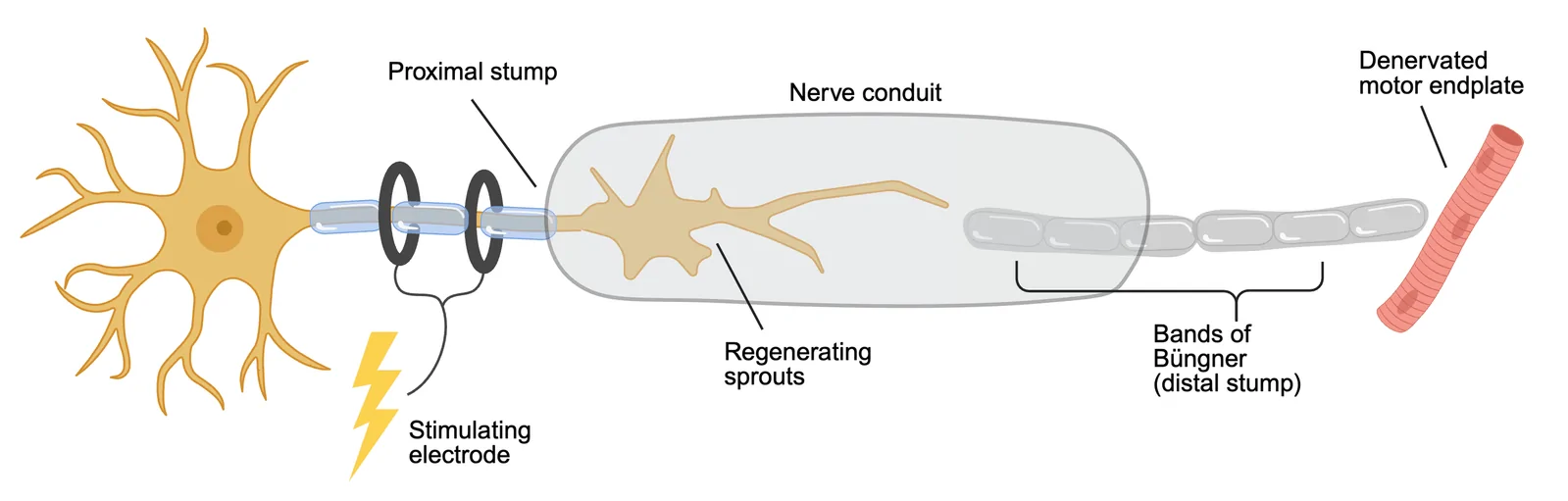
The repaired peripheral nerve during axonal regeneration Longitudinal schematic approximately two to three weeks after repair. The proximal axon remains myelinated. Distal to the injury, Wallerian degeneration has removed the axon and its myelin, leaving Schwann cells aligned end to end within surviving endoneurial tubes as bands of Büngner. Regenerating sprouts advance from the proximal stump through the conduit at approximately 1-3 mm per day and have not yet reached the denervated motor endplate. A stimulating electrode is positioned proximal to the repair site. Electrical stimulation acts at three sites: the cell body, where it elevates cyclic adenosine monophosphate (cAMP) and sustains growth-associated gene expression; the repair site, where it accelerates sprout crossing; and the distal stump, where it maintains neurotrophic support and delays Schwann cell senescence. Labels: stimulating electrode, proximal stump, regenerating sprouts, nerve conduit, bands of Büngner, distal stump, denervated motor endplate. Created in BioRender. Hudson, L. (2026); https://BioRender.com/ft5ga24.

Future directions and research gaps 

Despite growing mechanistic and preclinical evidence supporting ES as an adjunct to peripheral nerve repair, significant gaps remain before this strategy can be routinely integrated into clinical practice for median nerve injuries. Current literature is largely derived from animal models or small human studies that evaluate ES in isolation, often under controlled conditions that do not reflect the complexity of real-world nerve trauma [28]. A recent systematic review highlights the gap between preclinical promise of ES and clinical evidence, with its finding of only seven clinical studies currently existing, representing a total of 229 patients [28]. This limited pool of evidence represents a limitation in the field and for clinical use [28].

As a result, translation to severe median nerve injuries, particularly those associated with complex injuries such as electrical burns or extensive soft tissue compromise, remains limited.

Integration With Reconstructive Strategies

Few reports evaluate ES in combination with conduit-based reconstruction and vascularized soft tissue coverage, despite a strong biological rationale for synergy among these approaches [13]. Median nerve injuries frequently involve long regeneration distances, ischemic environments, and extensive fibrosis: conditions in which isolated interventions are unlikely to succeed [17, 20]. Vascularized nerve grafts are indicated for large nerve gaps and poorly vascularized recipient beds, yet their combination with ES has not been systematically studied [13]. 

Future investigations should prioritize multimodal treatment paradigms that combine structural guidance, biological stimulation, and optimized tissue environments to more closely reflect clinical scenarios.

Standardization of Stimulation Protocols

Considerable variability exists across studies with respect to stimulation timing, frequency, duration, and delivery method [28]. A systematic review found that stimulation frequency had a median of 20 Hz, but intensity varied from 3 to 30 V and pulse width ranged from 0.1 to 1.007 ms, with session duration being either 20 minutes or one hour [28]. While brief (one hour) low-frequency (20 Hz) ES applied acutely after repair emerged as a promising parameter for different injury types, reconstruction strategies, and patient populations remain undefined [17, 28]. However, due to the limitations of a narrative review and lack of sufficient evidence, no single protocol or quantitative findings can be reported as optimal.

Mechanisms underlying conditioning ES (delivered before injury) versus postoperative ES (delivered after repair) are distinct, with conditioning ES accelerating the intrinsic axonal regeneration rate and postoperative ES accelerating crossing at the injury site [29]. Prospective studies designed to systematically compare stimulation protocols are essential to establish reproducible and clinically actionable guidelines. 

Median Nerve-Specific Outcomes

Median nerve-specific outcome data are scarce, constraining interpretation of existing evidence. A systematic review of clinical applications identified only seven studies with 229 patients, all involving upper extremity injuries, but most aggregated results across multiple peripheral nerves despite substantial differences in fascicular complexity, functional demands, and regeneration distances [28]. Given the median nerve's critical role in hand dexterity, sensate pinch, and occupational function, future trials should include nerve-specific analyses and outcomes tailored to hand performance rather than generalized motor recovery alone.

Robust, standardized functional outcome measures are also needed. While electrophysiologic data and muscle reinnervation metrics are valuable, patient-centered outcomes such as grip strength, fine motor coordination, sensory discrimination, and return to functional independence are essential for determining clinical relevance [1]. Long-term follow-up is particularly important to assess whether early acceleration of regeneration translates into durable functional gains [20]. The current literature demonstrates that ES improves functional index, muscle mass, muscle force, conduction amplitude, and axon count, but whether these improvements translate to meaningful clinical outcomes over years remains unclear [28].

ES in High-Voltage Electrical Burn Injuries

The role of ES in the setting of high-voltage electrical burns remains largely unexplored. These injuries introduce unique pathophysiologic challenges, including microvascular thrombosis, progressive neural injury, and altered tissue conductivity, that may both complicate and potentially enhance responsiveness to bioelectric therapies. Focused investigation into how ES interacts with electrically injured tissue may reveal novel opportunities for targeted intervention in this high-risk population.

Taken together, these four gaps point to a common conclusion. ES represents a promising adjunct to median nerve repair, but its greatest potential likely lies within integrated reconstructive strategies rather than standalone application [17].

Carefully designed prospective studies that incorporate conduit reconstruction, soft tissue coverage, standardized stimulation protocols, and meaningful functional outcomes are needed to define its role in complex nerve injuries and guide future clinical adoption.

## Conclusions

Median nerve injuries remain among the most disabling peripheral nerve conditions due to their mixed motor and sensory architecture, long regeneration distances, and the narrow therapeutic window before irreversible distal stump and end organ deterioration occurs. While contemporary microsurgical techniques restore anatomic continuity, further advances are needed to recreate the biological environment required for consistent functional recovery.

The evidence reviewed here supports a multimodal rather than a single-modality approach. ES has emerged as a biologically rational adjunct that augments the regenerative capacity of foundational surgical repair. Preclinical and limited clinical evidence suggests that brief low-frequency stimulation applied early after repair improves regeneration. Conductive conduits and bioengineered scaffolds provide structural guidance, while vascularized soft tissue coverage addresses ischemia and fibrosis, particularly in electrically injured extremities. What the field lacks is not biological rationale but standardized stimulation protocols, median nerve-specific functional outcomes, and prospective trials evaluating these adjuncts in combination.
